# Language in Preterm Born Children: Atypical Development and Effects of Early Interventions on Neuroplasticity

**DOI:** 10.1155/2019/6873270

**Published:** 2019-02-25

**Authors:** Charlotte Vandormael, Lucie Schoenhals, Petra S. Hüppi, Manuela Filippa, Cristina Borradori Tolsa

**Affiliations:** Division of Development and Growth, Department of Child and Adolescent, University Hospital, Geneva, Switzerland

## Abstract

Predicting language performances after preterm birth is challenging. It is described in the literature that early exposure to the extrauterine environment can be either detrimental or advantageous for neurodevelopment. However, the emphasis mostly lies on the fact that preterm birth may have an unfavorable effect on numerous aspects of development such as cognition, language, and behavior. Various studies reported atypical language development in preterm born children in the preschool years but also in school-aged children and adolescents. This review gives an overview of the course of language development and examines how prematurity can lead to atypical linguistic performances. In this paper, we mainly focus on environmental and neurophysiological factors influencing preterm infant neuroplasticity with potential short- and long-term effects on language development. Further research, however, should focus on examining the possible benefits that early exposure might entail.

## 1. Introduction

Preterm (PT) birth is a phenomenon that affects a large and variable group of newborns due to its many underlying causes. According to a 2010 estimation by the World Health Organization, approximately 15 million babies are born PT, worldwide, each year [1]. Considering this large number, a broad amount of studies have been performed to examine the consequences of prematurity on development. Within the neuroconstructivism framework approach, the basis of cognitive development can be characterized by mutually induced changes between the neural and cognitive levels. Thus, PT infants' neurodevelopment is constrained by underlying brain structures which are, in turn, affected by experience-dependent processes [2]. This led to a definition of atypical rather than delayed development in the PT population (for a review, see [3]). In particular, in the first weeks of life, sensory development and behavior of the PT infant are negatively affected by neonatal characteristics and morbidities, the stressful environment of the neonatal intensive care unit (NICU), and social factors which may influence later neurodevelopment leading to complications such as motor delays, global cognitive impairment, visual perception problems, executive functioning deficits, and learning difficulties in school [4–6]. More specifically, children born PT show an increased risk for behavioral and attention difficulties [7, 8]. Furthermore, many studies have found a higher degree of language and social communication problems in PT-born children compared to full terms [9–12]. Deficits in expressive language, receptive language, word retrieval and short-term auditory memory were found [13]. In a meta-analysis performed by van Noort-van der Spek et al., which comprised of 17 studies on language development in PT children, it was discussed that even in the absence of major disabilities, very PT (VPT) survivors show difficulties in simple and complex language functions. This latter, involving higher-order cognition and highlighting the central role of cortico-cortical white matter tracts connectivity, might be a more useful indicator of the developing brain plasticity and of language functioning in PT children than simple language function. For complex language functions, in fact, PT's difficulties may even increase while growing up [14]. The extent of deficits a PT-born child may endure in life is associated with the complex interaction between multiple biological and environmental constraints following PT birth that occurs during a critical period of brain development and thus leads to atypical development [2, 3]. Studies examining environmental and biological factors as predictors of language skills in this group of children have been conducted [15, 16]. Stipdonk et al. concluded, in their very recent review, that language difficulties in the PT population are a consequence of an atypical brain connectivity between several brain regions, such as the cerebellum, corpus callosum, and arcuate fasciculi [17]. Nonetheless, it is essential to note that not all children born PT function lower than their peers born at term. In this paper, atypical language development and evidence of neuroplasticity during early development will be discussed in more detail.

## 2. Normal Language Development

### 2.1. Auditory System

Prior to the development of language comprehension and speech production, the auditory system has to develop. Already very early in gestation, the fetus's auditory system is formed. Between the 23rd and 25th weeks of pregnancy, important structures of the auditory system such as the cochlea are already in place. After 26 weeks of gestation, hair cells in the cochlea become fine-tuned for specific frequency bands, converting acoustic signals into electrical stimuli and forwarding them through the auditory nerve to the auditory cortex in the brain. Therefore, between the 26th and 30th weeks, the fetus is able to detect and react to sound stimuli [18]. This fine-tuning process takes place in the uterus where both internal (e.g., respiration, heart rhythm, and digestion) and external sounds (e.g., voices and music) can be perceived. Both types of sounds stimulate the auditory system by means of bone conduction, meaning that sounds are conducted to the inner ear through the skull. The observed frequencies are distributed tonotopically as on the basilar membrane in the cochlea, making the uterus the ideal place for auditory maturation as it acts as a low-pass filter, protecting newly developed hair cells from potentially harmful high-pitch tones. At the same time, high-frequency areas on the membrane will develop and the fetus can perceive human speech sounds (e.g., intonation, pitch, and intensity). Perceiving these high frequencies (+2 kHz) will enable later language processing. After the 30th week of gestation, the auditory system is mature enough to detect complex sounds and distinguish different phonemes in speech [18, 19].

### 2.2. Early Language Processing

Because hearing is functional during the last trimester of gestation, it is of interest to know whether and how these immature cortical circuits process speech. In utero, information about prosody and rhythm of the mother's speech is led to the fetus' inner ear by means of bone conduction through which they have the opportunity to learn about properties of their native language. Perceiving this speech signal is sufficient enough to shape an infant's phoneme perception prior to birth [20]. In addition, a clear difference was reported in the response to familiar versus unfamiliar language offers, showing that even before birth, the brain is being tuned to its language environment [21]. Furthermore, after only a few hours of postnatal exposure, neonates respond specifically to speech [22] and are able to discriminate between different prosodic patterns [23]. Brain networks sensitive to phonemes and voices are present at the very onset of cortical organization allowing the brain to already discriminate between small differences in speech syllables. This cortical activation during discrimination is not solely limited to primary auditory areas but also involves more inferior frontal regions [24]. Using noninvasive neuroimaging studies, speech-processing right after birth can be assessed. Stronger responses in the left temporal areas can be found when sentences are heard in the mother language whereas they are weaker when these same stimuli are played backwards, therefore erasing prosodic specificities of the mother tongue [25]. In 3-month-old infants, dominance in the left temporal areas for both forward and backward speech, with more activation in the left angular gyrus for forward speech, was found [26]. Minagawa-kawai et al. [27] also described a clear left lateralized cerebral basis for speech processing in 4-month-olds. Hence, it can be concluded that even early in infancy, there is a neural precursor of functional organization in the brain.

### 2.3. Later Language Processing

**Figure 1:**
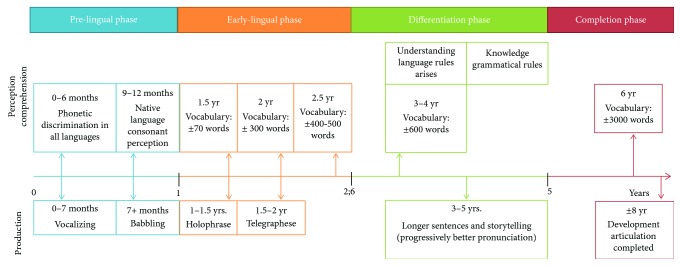
This figure shows the four language developing phases and the changes that occur in speech perception/comprehension and production in typically developing children during their first years of life.

Language acquisition after birth is made possible through the interaction between structural characteristics of the mother language and language offerings in the environment (child-directed speech). The overall process can be seen as a set of language skills that continuously grows. An infant learns to interact with the environment by producing sounds, actions, and behaviors. Different phases can be distinguished such as the prelingual phase (from birth to 12 months of age) when an infant starts vocalizing and babbling. Second is the early-lingual phase (from 1 to 2.6 years of age), during which a child shows signs of word comprehension and starts producing isolated words and short, telegraphic sentences. Next, in the differentiation phase (from 2.6 to 5 years of age), grammar starts to develop and sentences become more complete. Finally, during the completion phase (starting from 5 years of age), bases acquired in the preceding phases are further elaborated, for example, by developing reading and writing skills through education [28] (Figure 1).

## 3. Differences in Language Development between Full-Term and Preterm Born Infants

### 3.1. Prelingual Phase

In the first year of life, development of receptive language is crucial. During this period, the infant will learn to understand the mother language and how to respond accordingly. A prerequisite for this development are language offerings in the infant's environment (e.g., IDS (infant-directed speech)) and a correct-functioning hearing organ. In order to understand and produce speech, the infant needs to listen to a caregiver. Prelingual skills such as vocalizations, eye movements/gazes, gestures, and shared attention with a parent or caregiver are an important part of that process. During the first months of life, an infant establishes an infant-caregiver relationship by using eye contact, smiling, producing sounds, etc. [12]. Thus, speech perception is not solely an auditory process and the ability to detect auditory-visual matches in speech is already present at a young age [29]. Indeed, newborns prefer, for example, to look at the mother's face over a stranger's one when listening to the mother's voice [30]. Around the age of 6 months, infants can pay attention to the visual characteristics of speech, and as of 8 months they are able to observe auditory and visual characteristics of speech at the same time [31]. This skill is important during phonological development. Hence, infants benefit from a rich auditory and visual environment early in development [32]. However, for infants born PT who reside in the NICU, full-time parental presence and speech offerings in the environment are not always the case. Dysfunctions in early social communication with the parents, due to long periods of separation in the NICU, can have negative consequences on the communication between the infant and the caregiver [33]. The influence of PT birth on specific social experiences will be discussed later on in this review.

By listening to speech, an infant becomes more sensitive to characteristics of their native language, while during the second half of the first year of life, those of other languages disappear [34]. In typical development, children begin to enhance their native language discrimination abilities through the ages of 6 to 12 months, when the brain tunes itself to native phonemes and decreases the ability to discriminate between nonnative phonemes [35]. Jansson-Verkasalo et al. [34] studied these discrimination abilities in PT-born infants, focusing on the discrimination of two Finnish native phonemes and one native versus a nonnative phoneme in 6-month-old VPT born infants and full terms. No significant difference was found between the VPT and full-term groups at 6 months of corrected age when discriminating between the native and nonnative phonemes. However, between 6 and 12 months of age, the full-term group's response to nonnative stimuli decreased in relation to their response to the native phoneme. This typical decrease in nonnative vowel discrimination was not found in the VPT-born group as they continued to respond to the nonnative vowel. Additional research performed by Peña et al. [36] reported that neural maturation and not duration of exposure per se was a relevant factor for phoneme discrimination. Only at 9 months of age, PT-born infants performed at the same level as full-term controls (4 months of age). This finding is supported by later research concluding that the shaping of phonological representations by the environment is constrained by brain maturation factors [37]. At a later stage, a child develops phonological awareness to distinguish between phonemes and syllables and build different phonemic representations. More specifically, the awareness that individual sounds are the building blocks of words, e.g., “cro - co - dile,” allow a child to divide words into syllables, recognize and use rhymes, form phonemes into syllables and words, and identify the beginning and ending sounds of a word. Phonological awareness is an important prerequisite as it is the building block for future reading skills and vocabulary size [38]. Even without distinct brain damage, vocabulary and grammar difficulties were found throughout the first years of life in PT-born children and may even persist up to the school years as language competencies continue to be affected by weaker phonological awareness skills [39].

### 3.2. Early-Lingual and Differentiation Phase

After the first year of life, when the foundation of language comprehension has been laid, the infant will start to experiment more with spoken language. Expressive language involves learning to pronounce speech sounds and engage in communication. The expressive aspect of language can be subdivided into different skills, (1) lexicon/semantics: vocabulary, learning the meaning of words and (2) morphosyntax: grammatical development. A child learns to understand changes of word forms in different syntactic contexts and learns how to form correct sentences. A child will develop his or her vocabulary around the second year of life. On average, a child has acquired about 200-400 words at this age. Possessing an early lexicon has a highly predictive value for later language skills. In PT children, a linear relationship was found between gestational age at birth and later language outcomes. The lower one's gestational age at birth, the smaller the vocabulary size and quality of word use [40]. When comparing PT children, without any major cerebral damages, with their full-term peers, differences in linguistic development can be assessed [41]. Stolt et al. [42] examined the difference between the lexicon size of PT-born children in comparison to full-term peers at 2 years of age, but no significant difference was found. Contradictory results however were found, when PT and full-term born children were divided into three age groups (18-24 months old, 24-30 months old, and 30-36 months old). Both the PT and full terms showed an expansion of their expressive lexicon with increasing age. However, the lexicon of PT-born children was significantly smaller than those of their full-term peers [43]. Later, around 12-18 months of age, grammatical knowledge starts to develop. An experiment performed by Kunnari et al. [44] showed early delays in grammatical development in PT-born children by studying spontaneous speech samples at the age of 2. Results showed no difference in vocabulary size between the groups, but the maximum sentence length was significantly shorter in PT. Secondly, Stolt et al. [45] showed that VPT children, at the age of 2, had weaker grammatical skills than a full-term control group. However, when considering their lexicon size, less significant differences were found between both groups [42]. So, even though a delay in grammatical development was found in the VPT group, when taking lexicon size into consideration, it still develops in a similar manner as the full terms.

### 3.3. Completion Phase

As the child grows older, he or she will master the language better and start forming longer sentences. The meaning of words becomes better understood, and they can be formulated more accurately. This development is stimulated by education and acquiring reading skills. Reading requires good cognitive and intellectual development and is an essential skill for later academic purposes. Several prerequisites have to be established in order to learn how to read, i.e., phonological awareness, speech perception, and verbal skills. Each of these abilities contributes to the acquisition of two processes that are essential for becoming literate: firstly, decoding (single word reading) in which words are extracted from the mental lexicon, and secondly, word comprehension (semantics). In a meta-analysis performed by Kovachy et al. [46], fourteen studies assessing reading abilities in PT-born children between the ages of 6 and 13 years showed significantly lower scores for both decoding and reading comprehension. Similar results were found when studying long-term effects of prematurity on reading skills [47]. Significant correlations were found between lexical production and reading comprehension and between phonological awareness and reading comprehension in the PT group. Thus, comprehension, lexicon, and grammar can be negatively affected by PT birth, which may lead to an atypical development in reading and writing.

## 4. In Children Born Preterm, Is the Language Deficit Specific or Linked to a Global Cognitive Delay?

Language development is not solely dependent on language processes but also depends on basic cognitive processes (e.g., memory, processing speed, and attention) [48]. As abovementioned, PT birth and the complex interaction it entails between biological and environmental constraints may alter the pattern of brain development across brain regions, leading to atypical trajectories that may result in a global deficit of neuropsychological functions. In PT birth, language disorders are more often described as a result of such a general cognitive deficit [49, 50]. Indeed, it was found that PT children show high levels of comorbidity between cognitive functions and language. This might be accounted for by their similar functional dependence and demands [51]. Ortiz-mantilla et al. [52] indicated that language disabilities in very PT children can be explained primarily by general cognitive deficits which originate from global disturbances in brain development rather than damages to specific regions. In a study performed by Wolke et al., a detailed assessment of cognitive and language functions was described in a large sample of extremely PT children and term controls at the age of 6. It was shown that extremely PT survivors performed significantly lower on language assessment compared to term peers but also scored lower on measures of general cognitive functions. When controlling for general cognitive performance, the authors did not observe specific language difficulties in this population [50]. A tight relationship was found between phonological working memory and grammar in VPT children [53]. Also, dysregulation of attention, a system closely associated with language, influences social interactions and a child's opportunities for language learning may decrease [54]. In order to decipher whether language difficulties may be linked to overall cognitive delay or specific difficulties, studies should use cognitive abilities as a control variable and also address specific aspects of linguistic development and processes. Moreover, identifying deficits in general cognitive processes may help in the early detection of children at risk for impaired language development [55, 56].

## 5. Why Might There Be a Difference in Development?

### 5.1. Neuroanatomical Factors

Several recent studies have shown that cerebral abnormalities associated with PT birth may be a substantial determinant of cognitive and language development. Therefore, identifying predictors of development disorders through neuroimaging studies should help improve our knowledge.

#### 5.1.1. Atypical Functional Brain Organization

Variations in cognitive, language, and speech development are likely to be the result of underlying abnormalities in the brain associated with functional organization outcomes. During normal development, language organization is extensive and bilateral in the infant's brain. Later, with increasing age, it becomes more lateralized in the left hemisphere. In healthy 3-month-old infants, mature cortical areas are active when processing language. By this time, speech perception is already left-lateralized with activity in the superior temporal gyrus and the angular gyrus [26]. In PT newborns, shortly after birth, on the other hand, an asymmetry in the areas surrounding the perisylvian fissure is found, suggesting that specific anatomical organization favors functional lateralization even before language exposure [57]; this includes a larger depth of the right-sided superior temporal sulcus and a left shift of the planum temporale. Advanced structural maturation of the left-sided frontotemporal dorsal pathway of language was shown in 1-4-month-old infants indicating an early presence of circuitry underlying phonetic processing [58]. Mürner-Lavanchy et al. [59] examined language organization in PT-born children compared to controls using neuropsychological assessment and an fMRI language task. At early school age, PT subjects showed an atypical bilateral language organization in the frontal-temporal regions, whereas at 11-12 years of age they revealed left-sided language organization resembling that of the full-term group. These findings might reflect a delay of neural language lateralization in children born PT. Furthermore, Zhang et al. [60] studied 24 VPT-born children in comparison to matched controls at 7 years of age. In the VPT group, a greater regional vulnerability in the superior temporal sulcus and cingulate regions, with an abnormal asymmetry in the right hemisphere, was found. Similar results were reported in a study comparing 16-year-old VPT-born children with matched controls using the Peabody Picture Vocabulary Test and resting-state fMRI. In the VPT group, a positive correlation was found between more left lateralization and better language scores, with more activity in the left angular gyrus and inferior parietal lobe. On the other hand, a negative correlation was found between right hemisphere lateralization and language scores. Thus, less activation in these right hemispheric regions would lead to better language scores. It was hypothesized that early interventions strengthening the altered network can be advantageous for PT [61]. However, future research is needed to monitor how these interventions lead to changes in connectivity over time. Overall, these results show a delayed and atypical neural specialization for language systems in PT born compared to full-term-born children.

#### 5.1.2. Structural Abnormalities

Approximately 50-70% of VPT born infants are affected by diffuse white matter abnormalities such as loss of white matter volume, corpus callosum thinning, and delayed myelination [62]. The presence and severity of these cerebral injuries increase the risk of later neurocognitive impairment in PT children. In a recent study, it was reported that the mean score on language tests at the age of 4 and 6 years significantly declined as severity of white matter abnormalities increased [62].

The corpus callosum plays a crucial role in the exchange of interhemispheric information. Thus, a deviation may be associated with weaker cognitive performances. A study, in which the relation between the corpus callosum regions and preverbal skills was assessed in 14-15-year-old born VPT using structural MRI and neuropsychological tests, found a negative effect of VPT birth on the development of the corpus callosum. More specifically, a decreased volume in the corpus callosum posterior areas was positively associated with lower verbal IQ and reduced verbal fluency scores. Overall, this study demonstrates the involvement of the corpus callosum in speech and language processes and describes an interhemispheric asymmetry [63]. Additional research in the same domain showed an altered brain structure in VPT adolescents which accounted for lower spelling and reading scores in this population [64]. A subsequent MRI study demonstrated the importance of the interhemispheric frontal and temporal connections to predict language impairment. Results showed that the combination of anatomical measures of the interhemispheric connectivity between the corpus callosum and the anterior commissure explained 57% of the variance in linguistic abilities [65]. Finally, Reidy et al. [66] demonstrated that white matter alterations occurring during the neonatal period were predictive of abnormal language performances in VPT-born children at the age of 7.

### 5.2. Postnatal Environmental Factors

Considering brain development is mostly shaped by early sensory experiences, exposure to language in the infant's environment is of utmost importance [67]. Since PT-born infants are exposed earlier to the environment outside of the uterus, one can wonder what the impact of this early exposure to the auditory environment has on the developing brain.

#### 5.2.1. Effect of Exposure to Auditory Stimuli in the NICU

When born prematurely, infants spend the first weeks or even months of their life in the NICU. During this critical period for development, they are deprived of the sounds they would otherwise be hearing in utero. As discussed earlier, the intrauterine environment allows the fetus to perceive low-frequency sounds in an attenuated fashion, ensuring the development of the auditory system [68]. However, when born PT, infants are prematurely led into a more invasive environment which can have profound effects on the auditory brain maturation and subsequent speech and language acquisition [18]. Although PT infants residing in the NICU are deprived of maternal sounds, they are not deprived of all auditory stimulation. Unlike in utero, the auditory stimulation available to the infant depends on the NICU environment they are residing in. Firstly, the NICU environment may be too loud for the infant to reside in. While being placed in an open room, they are exposed to unpredictable multiple high-frequency sounds (electronic/machines) and voices (e.g., parents, nurses, and doctors) which may prevent them from being exposed to meaningful and infant-directed language inputs. In addition, excessive exposure to loud ambient noises can negatively affect the infant's physiological stability (e.g., affect the cardiovascular and respiratory systems), which in turn may cause a risk for neurodevelopment [69]. Secondly, the environment can be too quiet when the infant is placed in an incubator that does not allow them to perceive language stimuli [70].

A study by Caskey et al. [71] showed that a larger range of language exposure in PT babies can have a positive effect on later language development in the first weeks after birth. They hypothesized that PT-born babies residing in the NICU would have higher cognitive and language scores if they were exposed more to adult talk. In this study, a positive correlation was found between the number of words heard during the first weeks of life and the language and cognitive scores of the Bayley Scales for infant development III at 7 and 18 months of corrected age. Similar results were found by Montirosso et al. [72] when comparing very PT-born infants residing in 19 different NICUs to FT controls. Infants residing in a high-quality developmental care unit (better infant pain management, improved control of external stimuli, and more parental involvement) showed better receptive language skills than those residing in low-quality developmental care. Hence, these studies support the view that exposure to adult talk in the NICU is associated with better language and cognitive and communicative development at an older age [71]. It can be concluded that early adequate exposure to language and sensory stimulation is of great importance.

#### 5.2.2. Dysfunctional Caregiver-Infant Relationship

For infants residing in the NICU, full-time parental presence is not the case. Dysfunctions in early social communication, due to long periods of separation in the NICU, can have negative consequences on an infant's behavior and emotional and physiological well-being. Moreover, an important aspect affecting language development in PT born children is the quality of the infant-caregiver relationship. Multiple studies show that when a child and their caregiver participate in quality interactions, language development will improve. Increased psychological stress experienced by mothers of PT infants has been linked to differences in the mother-infant interactions in this population [73, 74]. On the Care Index, a measurement index that assesses mother-infant interactions, mothers of PT infants who are affected by maternal depression and anxiety have been found to be more controlling or unresponsive when interacting with their child, compared to mothers of FT infants [73, 74]. On the other hand, maternal anxiety may lead to more intrusive behavior, in which mothers provide less sensitive and a more controlling style of parenting. Zelkowitz et al. [75] studied whether anxiety affects maternal interaction and leads to less optimal communication into the preschool years. During their stay in the NICU, mothers were tested using the self-report State-Trait Anxiety Questionnaire, which is commonly used to indicate caregiver distress. Later, in a 24-month follow-up period, free play between the mother and child was observed. Results showed that anxiety during time in the NICU leads to less sensitive and responsive interactions between mother and child. In return, children involved their mothers less during playtime [75]. In light of the importance parent-infant interaction plays in language development, early intervention targeting these disordered dyads in the PT population could be beneficial. When PT-born infants participated in a postdischarge intervention program and attended regular visits to a pediatric hospital, better scores were found on the BSID. Moreover, mothers in the intervention group showed more positive and sensitive interaction behavior towards their child [76].

## 6. Can Preterm Infants Benefit from Early Auditory Exposure?

Although most abovementioned findings suggest a negative impact of PT birth, some studies suggest that PT infants can also benefit from early natural auditory exposure. Already at 29 weeks of gestational age, PT infants possess the ability to process subtle changes in phonemes and voices [24]. Hence, they are able to encode acoustic properties in order to perceive and process speech offerings in their environment. Furthermore, Nishida et al. [77] demonstrated that the duration of extrauterine exposure is correlated with enhanced brain responses. A shorter latency of oxyhemoglobin measures, using near-infrared optical tomography, was found in the PT group in response to verbal stimulation. Plus, despite potential structural differences, functional changes in the PT brain occur for both auditory recognition (differentiate between voice vs. reversed voice) and language decoding [78]. Also, differences in activation for the discrimination of two voices (mother vs. nurse) were found [79]. In addition, several auditory-evoked potential studies showed no differences in central auditory pathway maturation [80–82] or even reported that exposure of PT infants to the extrauterine environment is associated with advances in development compared to full terms [83]. Peña et al. [84] showed that PT infants benefit from early exposure to a visual environment (face-to-face interaction); at the age of 6 months, they performed the same as full terms with the same chronological age displaying that exposure does positively impact the development of gaze following. PT birth does open not only the possibility of a natural increase of positive auditory exposure [72] but also the possibility to intervene earlier to enrich their auditory experience. As it has been shown for the tactile experience, in which massage intervention affects the maturation of brain electrical activity, favoring a process more similar to that observed in utero in term infants [85], early auditory interventions can impact the PT infant's brain development. In fact, despite immature auditory pathways, early auditory interventions may have a positive influence on the PT infant's brain development. For example, PT infants residing in an environment in which they are more exposed to maternal sounds (mother voice) show larger auditory cortices [67]. Moreover, music interventions in the NICU have been shown to promote early language development and induce functional connectivity between the auditory cortex and additional brain areas associated with music processing [86, 87].

An early intervention found to be effective is interaction through maternal speech and singing, showing favorable effects on an infant's physiological state such as heart rate, oxygen saturation levels, and respiration rate [88]. Furthermore, a meta-analysis performed by Filippa et al. [89] evaluating 15 maternal voice interventions in 512 PT infants showed that maternal speech has a supporting role in clinical outcomes such as physiological state, behavior, and neurological development. More specifically, early exposure to the maternal voice through bone conduction can support neurobehavioral outcome and auditory development [90]. Hence, it can be suggested that PT birth may not always result in negative effects on language development. They may even perform better in specific discrimination tasks in comparison to their full term peers and prematurity can constitute a precious window of opportunities for enriching the PT infants' sensory experience.

## 7. Conclusion

The aim of this paper was to provide a detailed review of the literature on language development in PT-born children and to examine how prematurity can lead to atypical linguistic performances. According to the research discussed, it can be concluded that during the first years of life, crucial for gaining adequate social and adaptive skills, language development can be affected by PT birth. In VPT, altered brain maturation, leading to atypical functional organization and structural changes, was associated with abiding language impairments. In addition, environmental factors such as a long stay in a NICU with underexposure to significant auditory stimuli and nonoptimal infant-caregiver interactions have been associated with weaker language outcomes. Several intervention methods were proven useful in promoting the parent-child relationship, resulting in better interactions which have positive effects on cognitive and language development of children born PT. Moreover, we described some evidence of beneficial effects from early exposure to language, voices, and music in PT children. Further research is needed to assess the influence of this exposure on language development more thoroughly.
